# Short-term upper limb immobilization and the embodied view of memory: A pilot study

**DOI:** 10.1371/journal.pone.0248239

**Published:** 2021-03-11

**Authors:** Jérémy Villatte, Laurence Taconnat, Christel Bidet-Ildei, Lucette Toussaint

**Affiliations:** 1 Département de Psychologie, Université de Poitiers, Centre National de la Recherche Scientifique, Centre de Recherche sur la Cognition et l’Apprentissage (UMR 7295), Poitiers, France; 2 Département de Psychologie, Université de Tours, Centre National de la Recherche Scientifique, Centre de Recherche sur la Cognition et l’Apprentissage (UMR, 7295), Tours, France; 3 Département des Sciences du sport, Université de Poitiers, Centre National de la Recherche Scientifique, Centre de Recherche sur la Cognition et l’Apprentissage (UMR 7295), Poitiers, France; CNRS - Université d’Aix-Marseille, FRANCE

## Abstract

The present study aimed to explore the contribution of the manual sensorimotor system to the memory of graspable objects. Participants in the experimental group underwent a short-term upper limb immobilization design to decrease arousal to their dominant hand. Such designs are known to elicit updating of sensorimotor representations and to hardened use of implicit motor simulation, a process that occurs when observing graspable objects. Subsequently, a free recall and a recognition task of graspable and non-graspable objects took place. We found slower recognition for graspable than for non-graspable objects in the control group, while no differences appeared for the immobilized group. Moreover, the recognition latency for graspable objects tended to be slower for the control than for the immobilized group. These results suggest that a time demanding reactivation of motor simulation is elicited when a graspable object is correctly recognized by control participants. The effect of immobilization could prevent this reactivation, leading to faster recognition. Hence, immobilization selectively affects graspable object memory, showing a close relationship with the manual sphere of the sensorimotor system. We suggest that recognition accuracy would probably be affected in cases of stronger disruption of sensorimotor arousal.

## Introduction

According to grounded cognition theory, memory is deeply rooted in sensorimotor functioning [1]. Considering Gibson’s theory of affordance [2], Glenberg [3] proposed that the memory of an object is driven by the action potential that the object offers to an organism. Similarly, the embodied models [4, 5] describe long-term memory encoding as integration of sensorimotor activation and retrieval as the reenactment of a particular pattern of sensorial, motor and emotional experiences. Memory traces are encoded as a pool of sensorimotor features integrated while experiencing the present situation. Retrieval corresponds to the online reenactment of this previously experienced sensorimotor pattern. Growing evidence supports this assumption and indicates that encoding, consolidation and retrieval processes rely, at least partially, on sensory and motor features of experiences [6–9]. Furthermore, this view is consistent with numerous results in cognitive neurosciences in which similar sensory cortices were found to be activated during experience and recollection of a particular episode (for a review see [10]).

As human cognition is based on various sensory and motor systems, their relative contribution to memory can be questioned. Moreover, it can be assumed that depending on the considered memory trace, all sensorimotor systems do not provide equally important information. For example, knowledge about graspable objects likely involves a strong contribution of information from the manual sensorimotor system while information about the olfactive system can be almost irrelevant in such cases. Graspable objects are of main interest for embodied cognition because of their close relationship with sensorimotor functioning. According to Gibson [11], an object can be considered as graspable if it has “an opposite surface, separated by a distance less than the span of the hand” or “a handle to afford grasping” in the case of large objects. Most of the time, the term “graspable” is used for unimanual objects, grasped with a power or a precision grip. Neuroimaging studies provide evidence that mere observation of graspable objects triggers activity in the motor pathway, from the premotor cortex to the hand [12–15]. On the behavioral side, the results obtained through the stimulus-response compatibility paradigm indicate a potentiation of manual motor activity when people need to process graspable objects [16–19]. These results are often interpreted as indicating a contribution of the sensorimotor system for cognitive tasks in which graspable objects are either perceived or processed in categorization tasks. Moreover, they can be related to implicit neural simulation of action. Thus, according to motor simulation theory [20], the motor system is engaged not only in overt motor activity but also in covert, simulated actions related to possible activity envisaged with various graspable objects.

To our knowledge, only a few studies have investigated the specific contribution of the manual sensorimotor system to the long-term memory of graspable objects. Furthermore, the results can be regarded as slightly inconsistent. Using a motor congruence paradigm, van Dam and colleagues [21] found an effect of overt motor activity on recognition of graspable objects. First, their participants learned words denoting objects usually associated with two different types of manual actions, twisting or pressing. They were subsequently asked to perform an intervening task that involved either pressing or twisting. Better recognition performance was found in the congruent condition (i.e., when the executed action was similar to the action associated with the objects previously learned) than in the incongruent condition (i.e., when the executed action differed from the action associated with the objects previously learned). This result emphasizes the contribution of the manual sensorimotor system to the memorization of graspable objects by showing a direct relationship between motor activity and memory trace formation. However, in a recent attempt to extend this finding, Zeelenberg and colleagues [22] failed to replicate the gesture congruency effect with a larger sample. Besides, the hypothesis that motor actions play a role in the memorization of object pictures and object names received no support from the experiment conducted by Canits Pecher and Zeelenberg [23]. They reported that motor compatibility had no effect on recall and recognition memory, although being present in a previous semantic categorization task.

In parallel with overt motor activity, motor imagery can also be among the numerous sensorimotor properties integrated and reenacted in long-term memory. Using a motor interference paradigm, Paulus et al. [24] demonstrated that motor simulation has a role in the acquisition of functional knowledge about unknown objects. In the same vein, Dutriaux and colleagues [25, 26] found evidence that motor simulation can favor objects memory, by manipulating hands position on the free recall of pictures and words referring to graspable objects. Their participants were asked to learn lists of items while adopting either a control posture (i.e., with the hands at rest on a table) or an interfering posture (i.e., with the hands crossed behind their back). The results showed a decrease in free recall performance for items referring to graspable objects in the interfering condition, whereas hands posture had no influence on non-graspable objects. This postural effect can be interpreted as a consequence of hardened activation of implicit motor simulation processes when the hands are not immediately available to engage in manual interaction. Note however that the effect of hand position disappeared for objects presented outside of the arm reaching space (i.e., in the extrapersonal space) compared to the participants’ peripersonal space [27].

To sum-up, although little evidence suggest that manual sensorimotor system has a selective role for the memory of graspable objects, the inconsistent results in the literature require further investigation. The aim of the present work is to provide evidence for this hypothesis using short-term hand immobilization, known to be an adequate method for undermining the functioning of the sensorimotor system. Because of use-dependent plasticity, peripheral changes elicit modifications in the central nervous system. It is well-known that cortical organization of a given body’s area are increased by stimulation [28, 29], the opposite being found in the case of sensory input deprivation produced by limb amputation [30] or weeks of limb immobilization [31]. In line with these results, even short-term upper limb immobilization (a few hours) causes functional changes in the central nervous system [32–34] and disturbs cognitive activity. More precisely, 24 hours of upper limb immobilization negatively affects tasks such as action verbs understanding [35], implicit action simulation [36, 37] and grasp representation which contain concrete information on actions [38]. Therefore, following the embodied approach of memory, we assumed that short-term hand immobilization can affect graspable object memory through its negative effect on motor imagery functioning. We hypothesized that 24 hours of hand immobilization would reduce manual sensorimotor stimulation with the consequence of causing markedly disturbed memory for graspable objects. More specifically, for the free recall task, we expected lower memory performance for graspable objects than for non-graspable objects in the immobilized group. No such differences were expected in the control group, i.e. for the participants who did not undergo the immobilization procedure [25, 26]. For the recognition task both accuracy and response latencies were recorded. The effect of sensorimotor deprivation could be highlighted in terms of accuracy (i.e., the number of correct recognitions/false alarms) and/or in terms of information processing speed, with longer recognition latencies for graspable objects in the immobilized group due to the slowdown of sensorimotor processes.

## Methods

### Participants

Sixty-nine French speaking university students (18–27; mean age = 20.7; 44 females) participated in the experiment. All of them were right-handed according to their results at the Edinburgh Handedness Inventory [39] (mean score = 80, SD = 19, control group = 80, SD = 20, immobilized group = 81, SD = 18). They had normal or corrected to normal vision and reported no medical history. The study is conformed to the Declaration of Helsinki and was approved by the ethical committee of the Research Centre on Cognition and Learning. The participants gave their written informed consent and received course credit or a €20 remuneration. They were assigned on a voluntary basis to the control group (n = 34, 23 females) or to the immobilized group (n = 35, 21 females). To control for difference in crystalized abilities, they completed the Mill Hill vocabulary test [40]. The results did not show any difference between the control (mean = 20.09, SD = 3,68) and immobilized groups (mean = 21.14, SD = 3.31; *t*_*67*_ = 1,25; *p* = .21).

### Material

One hundred and twenty-eight pictures of objects, half of them graspable (e.g., scissors, apple), and the other half non-graspable (e.g. sofa, elephant) were used as stimuli. Objects appeared on a light gray background. LEXIQUE database [41] was used to control for lexical frequency and length of the words referring to the pictures. Graspable and non-graspable objects were comparable on both factors [lexical frequency *F* (1,126) = .04, *p* = .85; length *F* (1,126) = 2.04, *p* = .16]. The set of pictures was distributed into two lists of 32 graspable objects and two lists of 32 non-graspable objects (S1 Appendix). The lists were equivalent regarding lexical frequency and word length (S2 Appendix). That is, no difference appeared between the two graspable objects lists [lexical frequency *F* (1,62) = 0.96, *p* = .32; length *F* (1,62) = 0.19, *p* = .66] or between the two non-graspable objects lists [lexical frequency *F* (1,62) = 0.01, *p* = .91; length *F* (1,62) = 0.53, *p* = .47]. For each participant, a list of both object types was randomly selected as the learning list, and the other as the distractive list.

### Task and procedure

Participants were tested individually in a quiet room, seated approximately 60 cm from the computer screen, with the experimenter present.

One day before the beginning of the experiment, a rigid splint (model DONJOY “Comfort Digit”, DJO, Surrey, UK) was fixed on the dominant (right) hand of the participants assigned to the immobilized group. The splint immobilized their hand, wrist and fingers. An immobilization vest restrained the movement of their right arm to ensure that they kept their hand at rest as much as possible for 24 hours. They were also instructed to wear actimeters on both hands to obtain an objective measurement (count/min) of their physical activity. Participants were instructed not to remove the splint and the actimeters and were allowed to remove the immobilization vest only for shower and bedtime. Because of technical issues with actimeters, physical activity recording was unavailable for 5 participants. A paired sample *t*-test performed on the remaining 30 participants showed that their level of activity was significantly higher for the left hand than for the right immobilized hand (mean counts left arm = 2668, SD = 771; mean counts right arm = 643, SD = 278; *t*_*29*_ = -18; *p* = <001). The immobilization vest and the splint were removed only before the very beginning of the experiment. Crucially, as soon as the splint was removed, participants were instructed to put their hand at rest on their legs and to inhibit any hand or finger movements to avoid sensorimotor awakening. Similar instructions were given to the control group.

**Fig 1:**
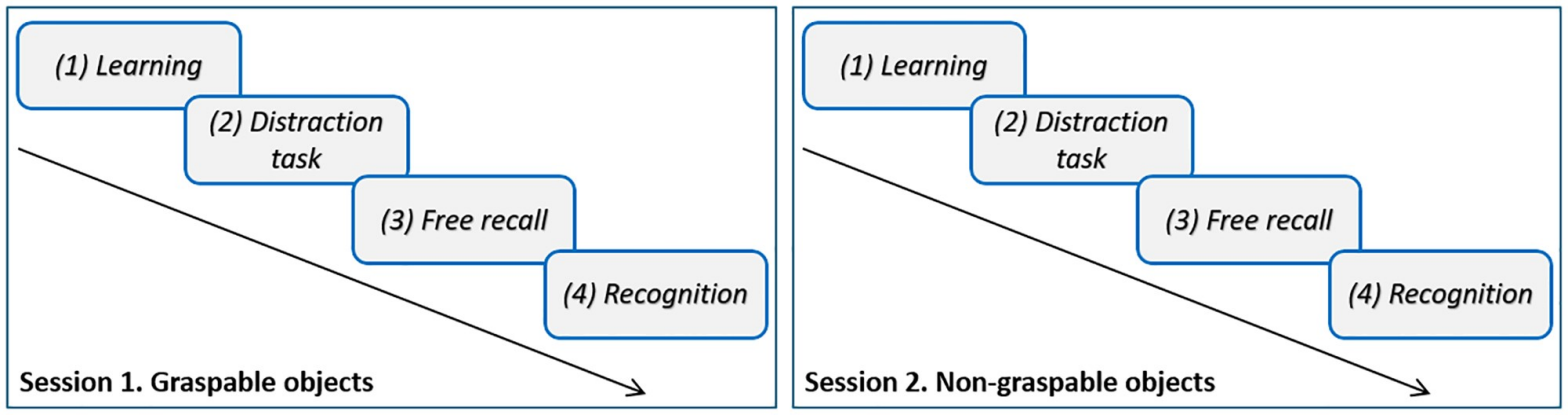
Experimental procedure; the session order was counterbalanced across participants.

The experiment was divided into two successive sessions. One session was a memory task on graspable objects, and the second was an identical memory task on non-graspable objects. The sessions order was counterbalanced across participants. Memory tasks within each session involved four different phases. During the *learning phase* (1), participants were instructed to memorize as many pictures as they could, in order to recall them later. Each picture was presented in a random order, for two seconds, preceded by a one-second fixation cross. As soon as the learning phase ended, participants performed a one-minute *distraction task* (2) with pairs of letter comparisons. The first letter was an upper-case, the second lower-case. The participants had to orally indicate whether the letters were the same (e.g., A a) or not (e.g., A b). It has been proposed that this distractive task decreases the risk of semantic interference [25, 26]. Then, the participants performed a *free recall task* (3). They were asked to orally recall as many items as they could, without time limitations, while the experimenter reported their answers on a data sheet. Free recall ended when participants explicitly indicated they had given all items they were able to remember. Last, was the *recognition task* (4), the 32 “old” pictures of the previously learned list were randomly displayed among 32 “new” pictures of the distractive list. In the *recognition task*, verbal answers were given using a microphone connected to the computer: a picture previously seen during the learning phase shall be associated with an “old” answer, a picture that was not presented during the learning phase shall be associated with a “*new*” answer. The E-prime 2.0 software package [42] was used to display the stimuli and to record recognition latencies. The experimenter wrote the accuracy of answers on a data sheet. When the recognition task was completed, session 2 began immediately, with the same phases realized on different object type (Fig 1).

## Results

### Data analysis

For both free recall and recognition tasks, analyses consisted of 2x2 ANOVAs performed with different groups (control/immobilized) as a between-subjects factor and object (graspable/non-graspable) as a within-subjects factor. Significant interactions were investigated further with a Newman-Keuls multiple comparison post-hoc test.

For the free recall task, we used the rate of correctly recalled objects (Number of correct recalls/number of learned items) to assess memory performance. For the recognition task, we computed both correct recognitions rates (i.e. number of correct “old” answers/number of previously learned items) and false alarms rates (i.e. number of incorrect “old” answers/number of new items) to assess memory accuracy. Regarding information processing speed, we used participants’ response latencies for correct recognitions. Moreover, we also ran a similar analysis on correct rejection latencies (i.e., “new” answer for a non-learned item). Although correct rejections do not allow for investigation of changes in memory processing, we assume they provide an interesting benchmark. Hence, a comparison of correct recognition latencies with correct rejection latencies is a way to assess whether immobilization influences memory for previously learned items or mere visual identification.

Because microphone recording involves participants speaking loud enough and with clear voice, 3.9% of the data were not available due to voice detection failure. Response latencies below 500 ms were excluded, as such rapid answers are likely to reflect anticipation errors. Moreover, response latencies beyond 4000 ms were also excluded as we assume they did not represent the normal time course of the recognition process. Eventually, such extreme values were very rare, less than 0.3% of the available dataset for correct recognition latencies and less than 0.5% for correct rejection latencies.

### Free recall & recognition accuracy

For the free recall task, analysis performed on correctly recalled objects (Table 1) indicated no main effect of group *F* (1,67) = 0.07, *p* = .80 and object *F* (1,67) = 0.001, *p* = .97. The interaction between group and object was also nonsignificant *F* (1,67) = 0.07, *p* = .79.

**Table 1:** Percentage of correctly recalled objects, correct recognitions, and false alarms as a function of objects (graspable vs. non-graspable) and groups (control vs. immobilized).

		Control (n = 34)	Immobilized (n = 35)
**Free recall**	*Graspable*	42 (8)	42 (12)
	*Non-graspable*	41 (9)	42 (11)
**Correct recognition**	*Graspable*	93 (8)	91 (8)
	*Non-graspable*	93 (7)	92 (7)
**False alarm**	*Graspable*	9 (7)	8 (7)
	*Non-graspable*	7 (6)	8 (9)

Standard deviations are in parentheses.

Regarding the recognition task, analysis performed on correct recognitions revealed no main effect [group, *F* (1,67) = 0.46, *p* = .50; object *F* (1,67) = 0.67, *p* = .41] or interaction [*F* (1,67) = 0.04, *p* = .84]. Similarly, no difference was found between false alarms for graspable and non-graspable objects *F* (1,67) = 1.09, *p* = .30. The main effect of group *F* (1,67) = 0.05, *p* = .82 and interaction *F* (1,67) = 0.52, *p* = .47 were also not significant (Table 1).

### Correct recognition latency

**Fig 2:**
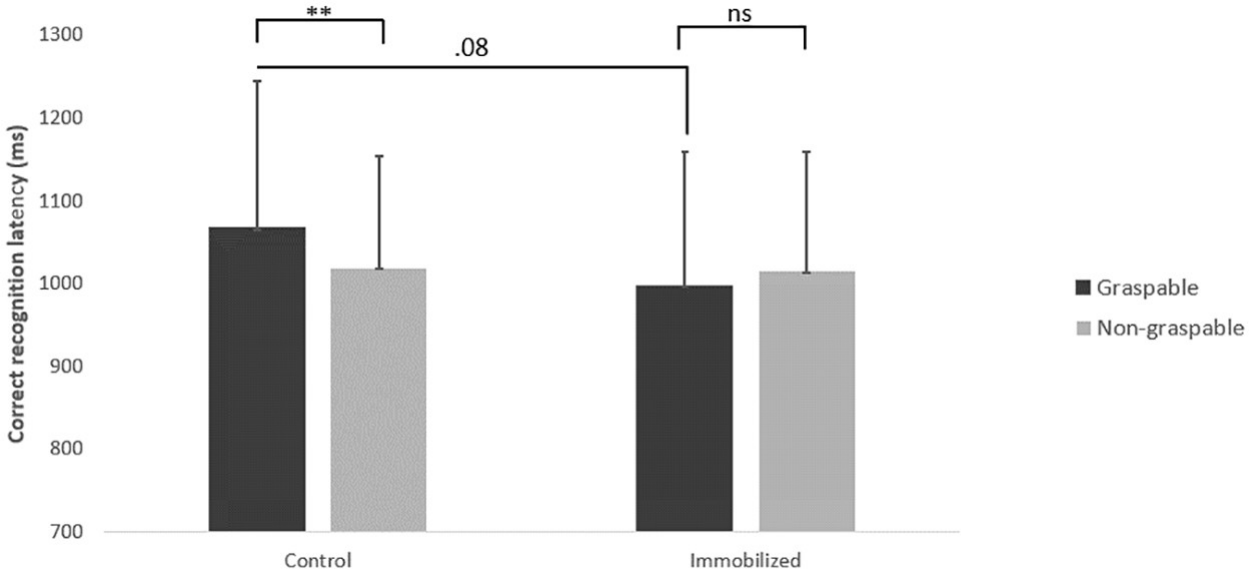
Correct recognition latencies (ms) as a function of group (control vs. immobilized) and object (graspable vs. non-graspable).

The results showed no main effects of group *F* (1,67) = 1.04, *p* = .31 or object *F* (1,67) = 1.53, *p* = .22, but the group x object interaction was significant *F* (1,67) = 7.28, *p* < .01, *η²p* = .10. Newman-Keuls post-hoc comparisons indicated that immobilized participants had similar hit latencies for graspable (M = 998 ms, SD = 135 ms) and non-graspable objects (M = 1016 ms, SD = 146 ms) (*p* = .30) while control participants were slower to recognize graspable objects (M = 1067 ms, SD = 178 ms) than non-graspable objects (M = 1019 ms, SD = 162ms) (*p* < .01). Furthermore, the effect of group on graspable objects was close to the significance level (*p* = .08) suggesting that immobilized participants tended to be faster than control participants (Fig 2).

### Correct rejection latency

Analysis yielded no main effect of group *F* (1,67) = 0,10, *p* = .75, or object *F* (1,67) = 0,08, *p* = .77. More crucially, the interaction between group and object was above the significance threshold *F* (1,67) = 1,09, *p* = .30 (Table 2).

**Table 2:** Mean correct rejection latencies (ms) for graspable and non-graspable objects.

	Control (n = 34)	Immobilized (n = 35)
*Graspable*	1179 (200)	1216 (248)
*Non-graspable*	1204 (250)	1201 (221)

Standard deviations are in parentheses.

## Discussion

The present work was designed to gather evidence for a selective implication of the manual sensorimotor system in the memory of graspable objects. Three types of measurements were used to assess memory functioning: free recall, recognition accuracy and recognition latency for both immobilized and control (i.e., nonimmobilized) participants. A rigid splint and an immobilization vest restrained the movement of the right upper limb of the immobilized participants over 24 hours. The immobilization device was removed only before the very beginning of the memory tasks. We assumed that updated sensorimotor representations along with motor imagery perturbation due to short-term hand immobilization [36, 37] could affect memory with lower performance for graspable objects than for non-graspable objects. By contrast, in agreement with the literature [25, 26], no difference between graspable and non-graspable objects was expected in the control group. The main results confirmed that memory functioning differed for control and immobilized participants. Surprisingly, the results highlighted slower recognition for graspable than for non-graspable objects in the control group, while no differences appeared for the immobilized group. Moreover, the recognition latency for graspable objects tended to be slower for the control than for the immobilized group. No difference between group appeared for free recall and recognition accuracy.

The results of the present experiment show that short-term upper limb immobilization modifies the functioning of recognition processes and specifically the recognition latency. This type of results confirms that the sensorimotor system plays a role in the speed of information processing required to correctly carry out the recognition task, i.e., to analyze whether the displayed object was seen during the previous learning phase. However, sensorimotor disturbance does not seem to affect the amount of information stored in long-term memory (i.e. the number of recalled items), unlike reported in other studies [21, 25, 26]. It could be that limb immobilization led to lesser disruption of implicit action simulation than the interfering posture (i.e. when participants had to keep their hand crossed behind their back) used by Dutriaux and collaborators [25, 26]. Short-term upper limb immobilization is effectively known to slowdown the sensorimotor system, but no effect has ever been observed in terms of performance accuracy [34–37]. Another possible explanation could be related to the items used in the experiments. A careful look at the objects used in our study shows that some graspable objects could be less associated with hand action than others. For example, the feather or the watch, although graspable, could activate the sensorimotor system to a lesser extent than the hammer or the eraser, which are functional objects [43]. The immobilization-induced effects expected for graspable objects could have been attenuated by some objects in the list which would have activated the sensorimotor system to a lesser extent. Other studies will be performed in the future to clarify this point.

At first, the effect of immobilization on recognition latencies seemed quite surprising. It appears that control participants needed more time to recognize graspable objects than non-graspable objects. This difference was not observed for immobilized participants. Contrary to our expectation, recognition latency for graspable objects (compared to non-graspable objects) was not slowed by upper limb immobilization but sped up. Although this result was not expected, it is consistent with the idea that implicit motor simulation elicited by graspable objects may have a role in memory but is undermined by sensorimotor deprivation. According to the embodied models [4, 5], memory encoding involves to gather a bunch of experienced sensorimotor primitives in a trace, while retrieval requires the reenactment of these sensorimotor primitives. Implicit motor imagery could be one of the numerous primitives encoded in a memory trace of graspable object [25, 26]. Therefore, the longer latencies for graspable objects compared to non-graspable objects in the control group could be due to the reenactment of implicit simulation processes, among various other sensorimotor features. Considering that non-graspable objects do not activate the sensorimotor system, their recognition requires one less element and can therefore be done more quickly than for graspable objects. On the other hand, the fact that immobilized participants showed no difference for both types of objects (graspable vs. non-graspable) suggests that implicit motor simulation information may not be involved, probably because immobilization disturbed its uses. Therefore, for immobilized participants, graspable objects recognition would not involve a supplementary reenactment (i.e. motor simulation) as compared to non-graspable objects. In other words, no more difference in the amount of primitives to reactivate would lead to no more difference in recognition latencies. This reasoning is consistent with the observation made by Toussaint and Meugnot [36] in a mental rotation task who reported that due to weakened motor imagery abilities linked to short-term upper limb nonuse, some participants are no longer able to use motor simulation processes. Arguably, a quite comparable phenomenon occurs in a memory task on graspable objects. Because motor simulation is impaired following short-term immobilization, it is no longer used to solve the task (i.e. not encoded or not reenacted). As other sensorimotor primitives are available for encoding and reenactment, participants are still able to carry out the task without impact on memory performance itself, and the change can be seen on response latencies only. Besides, the analysis of correct rejection latencies, required to assess whether immobilization only influences the time course of memory (i.e. only “old” items are concerned) or visual perception of the object (“old” and “new” items are concerned), strengthens our suggestion. No immobilization-induced effect appeared for correct rejection latencies. Consequently, this result suggests that the difference between control and immobilized groups in correct recognition latencies does not come from a mere perturbation of visual processing. Therefore, our study confirmed that sensorimotor processes may have a role in long-term memory, as previously reported [21, 24–26]. However, to our knowledge, the present experiment highlighted for the first time in the literature that diminished sensorimotor system efficiency due to limb immobilization may force the participants to ignore sensorimotor information to perform a memory task. Although this processing change did not affect the accuracy of memory (i.e., the number of correctly recognized objects), it cannot be excluded that larger sensorimotor deficits can have deleterious effects on the accuracy of the information in memory. Further studies will be required to clarify this point as well as to investigate whether sensorimotor processes play a role during encoding and/or retrieval of information in long-term memory. The immobilization protocol used in the present experiment influenced the whole memory task, whereas previous works were focused on changes introduced only during the encoding [25, 26] or consolidation [21] phases.

To conclude, this pilot study is the first to our knowledge suggesting a direct relationship between short-term upper-limb immobilization and graspable objects memory. Twenty-four hours of upper limb immobilization affected the recognition latencies but did not disrupted the success of the task (i.e., the number of correct responses). Possibly, immobilized participants kept their memory performances constant by switching from sensorimotor to other, pertinent cognitive processes. Does this mean that sensorimotor processes are not important for memory? This could be the case when items to memorize are sufficiently familiar. However, the sensorimotor processes could be decisive in the learning of new items and, more specifically of items requiring unusual actions for which motor simulation is not easy. This point will be taken into account in future studies.

## Supporting information

S1 Appendix Items used for the memory tasks. (DOCX)

S2 Appendix Items’ names and characteristics. (DOCX)
