# Supplementary figures and images for: Short-term upper limb immobilization and the embodied view of memory: A pilot study
Source: PLoS One. 2021 Mar 11;16(3):e0248239. doi: 10.1371/journal.pone.0248239 (PMC7951805; doi:10.1371/journal.pone.0248239)

S1 Appendix

Items used for the memory tasks

Graspable objects


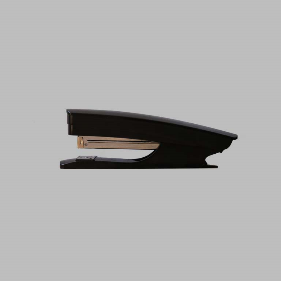

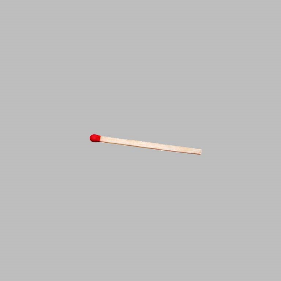

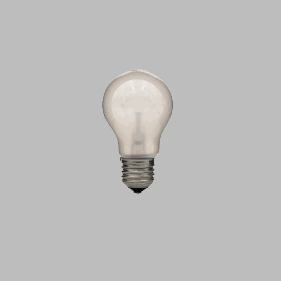

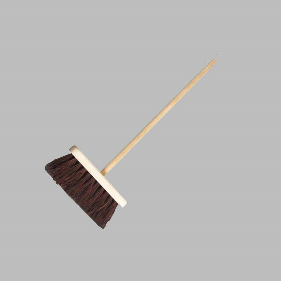


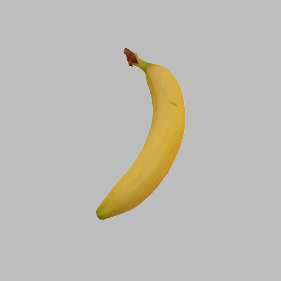

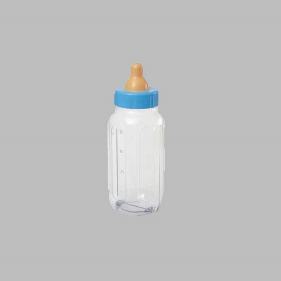

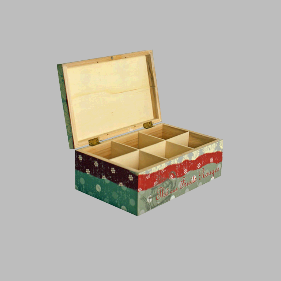

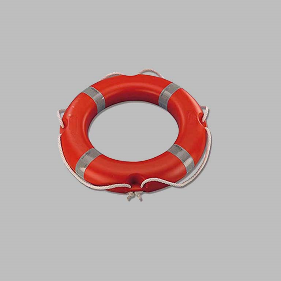


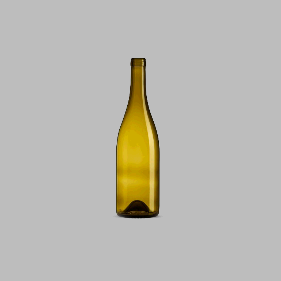

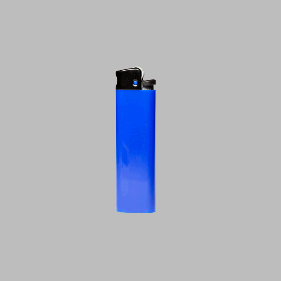

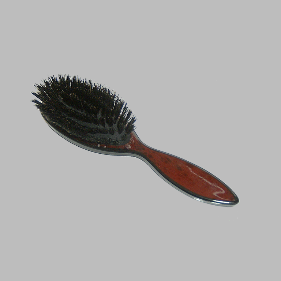

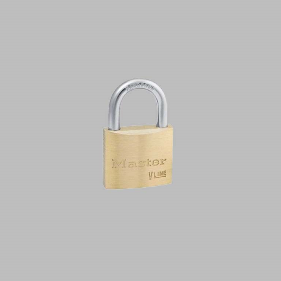


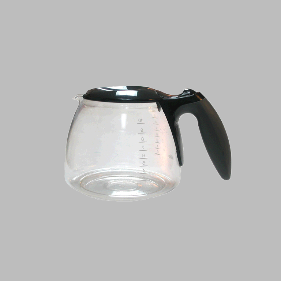

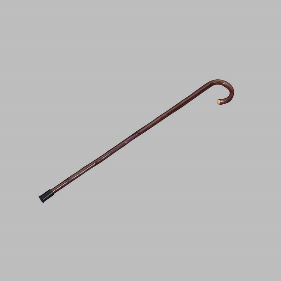

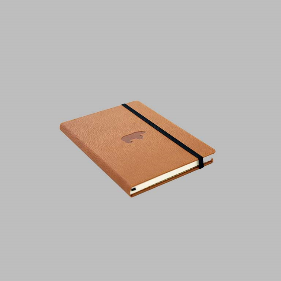

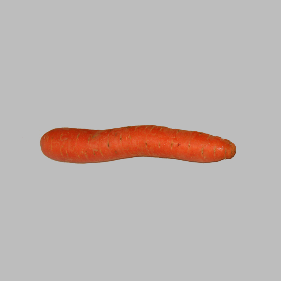


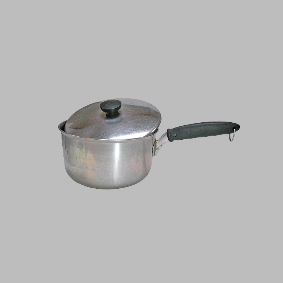

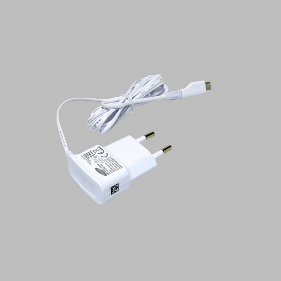

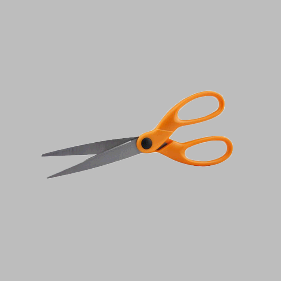

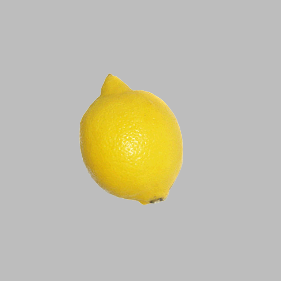


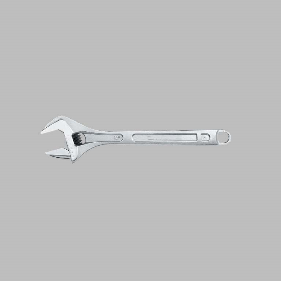

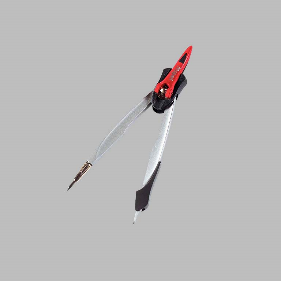

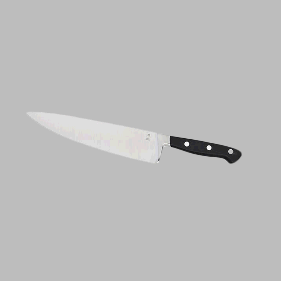

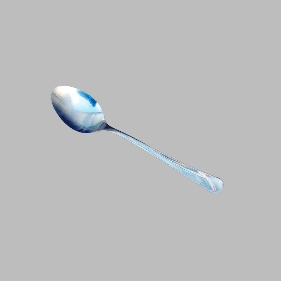


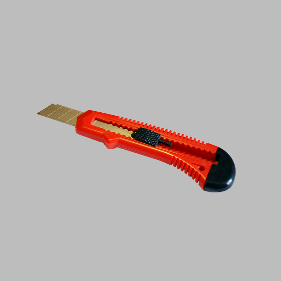

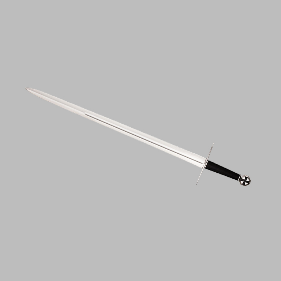

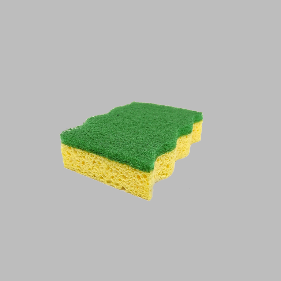

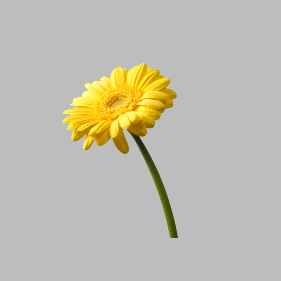


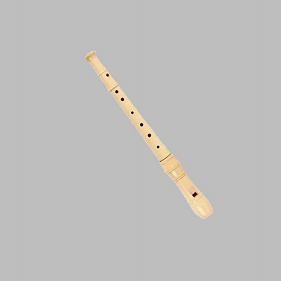

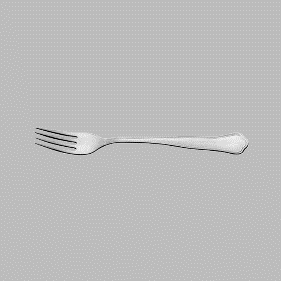

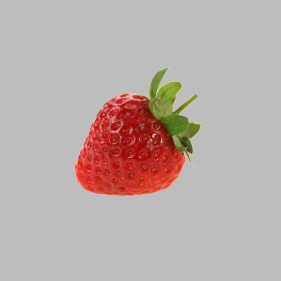

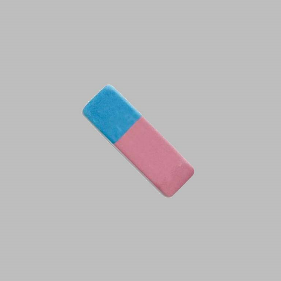


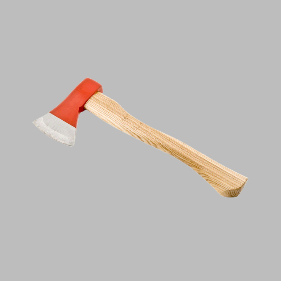

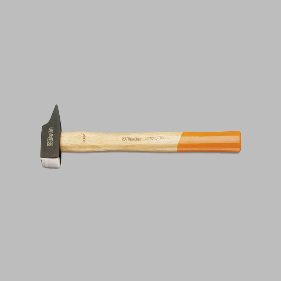

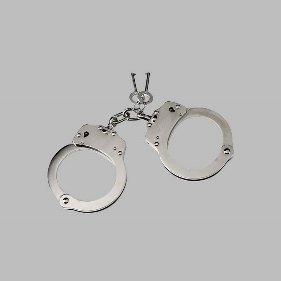

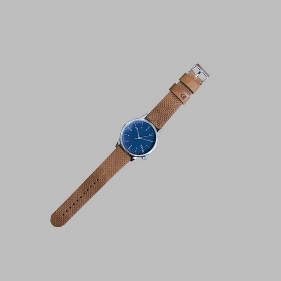


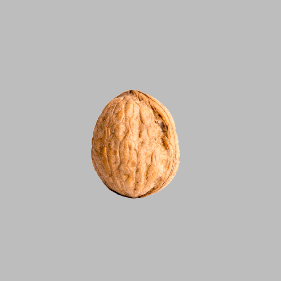

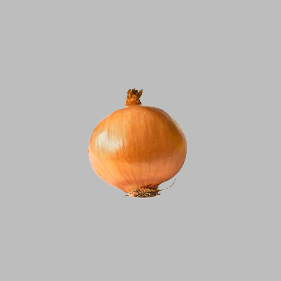

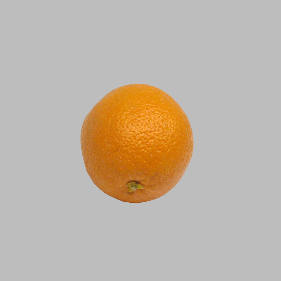

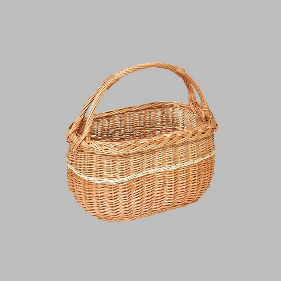


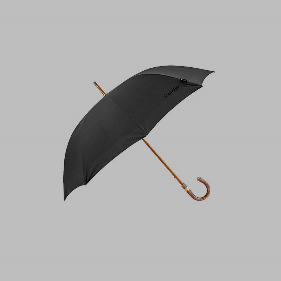

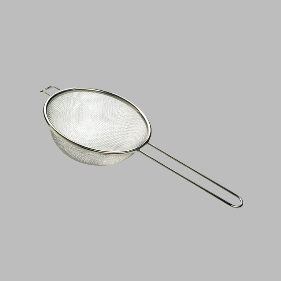

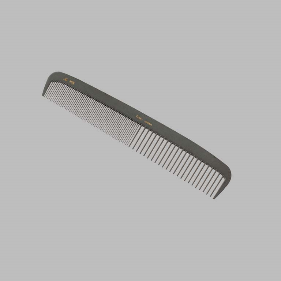

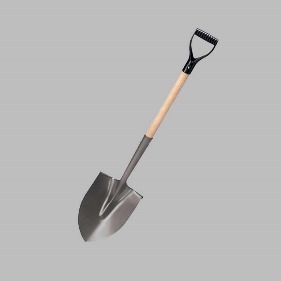


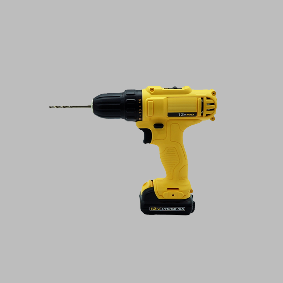

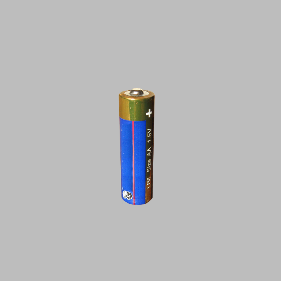

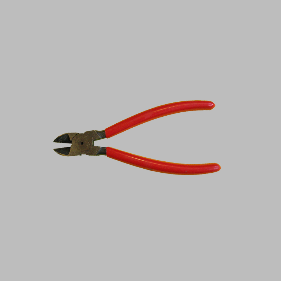

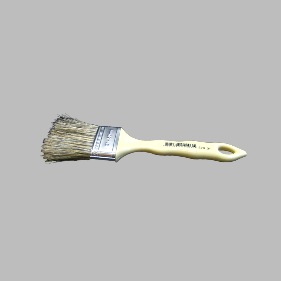


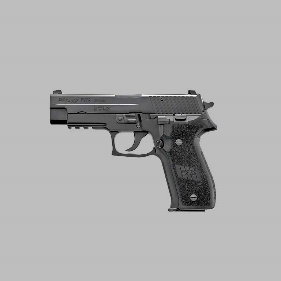

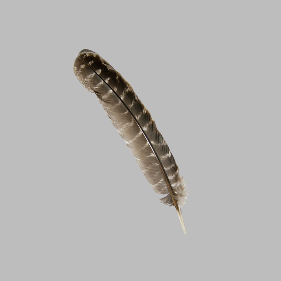

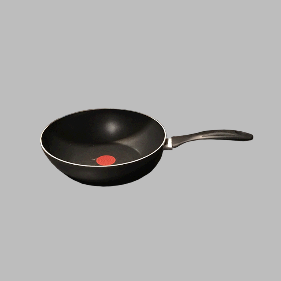

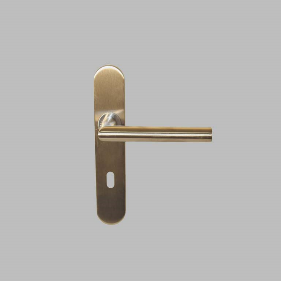


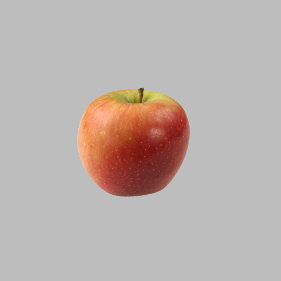

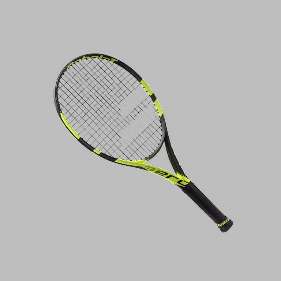

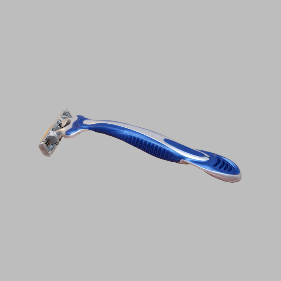

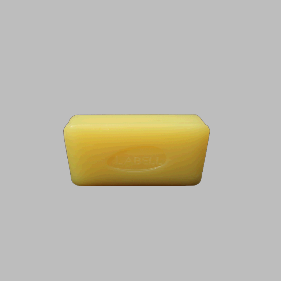


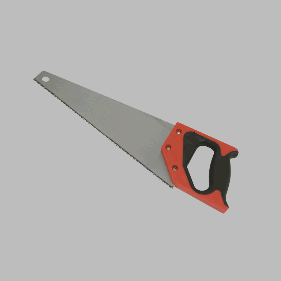

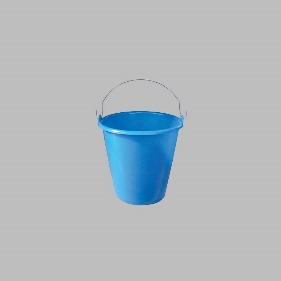

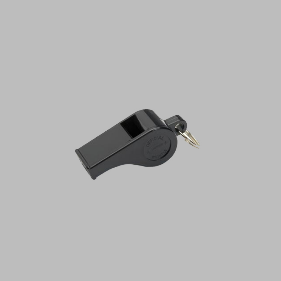

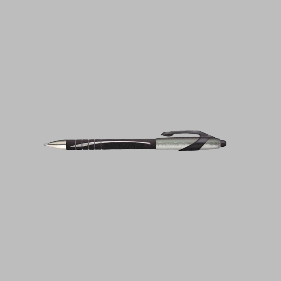


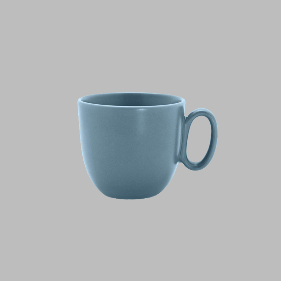

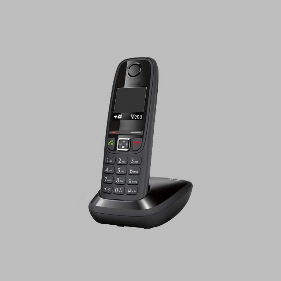

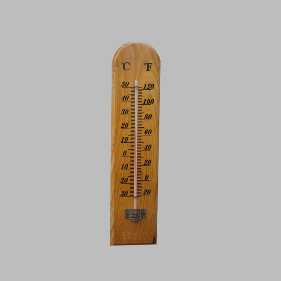

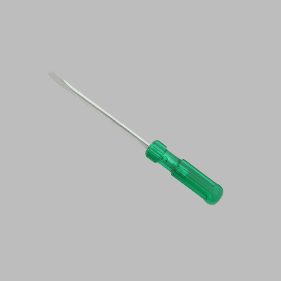


Non-graspable objects


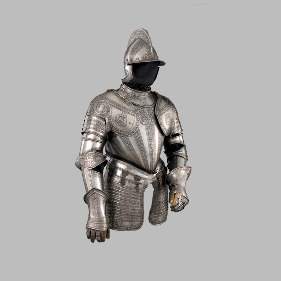

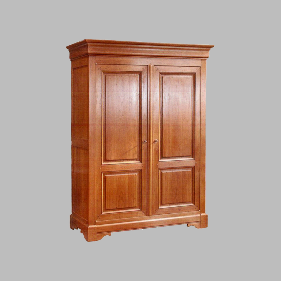

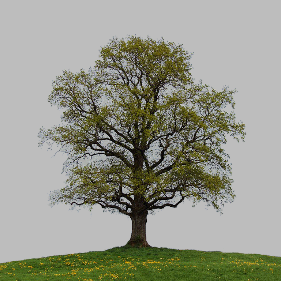

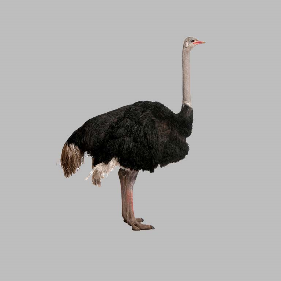


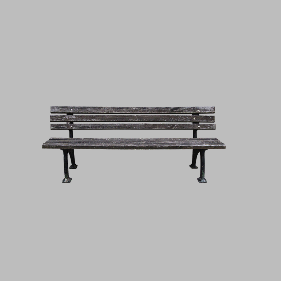

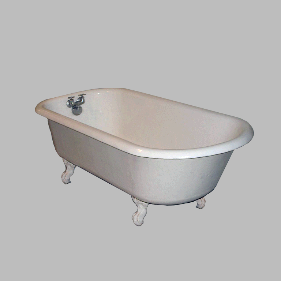

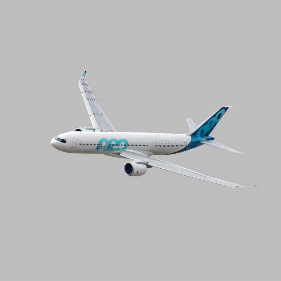

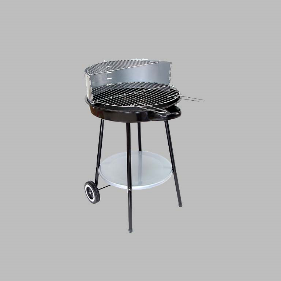


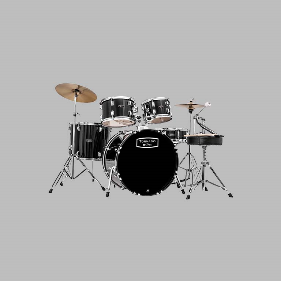

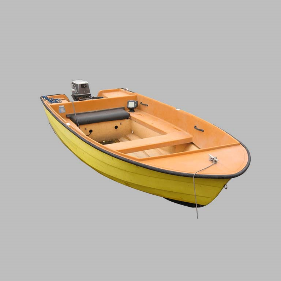

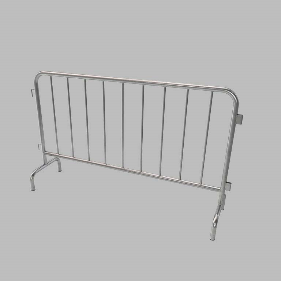

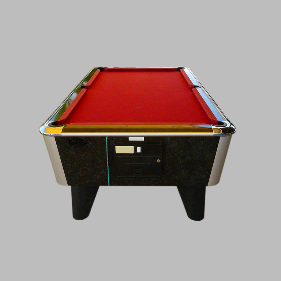


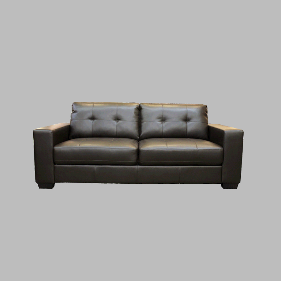

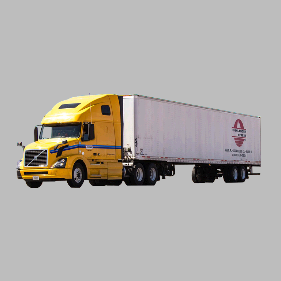

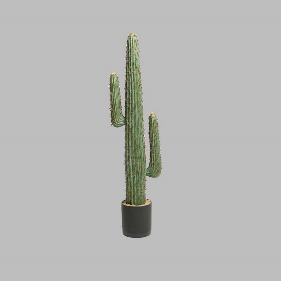

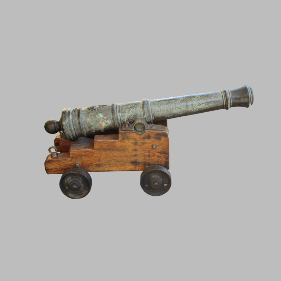


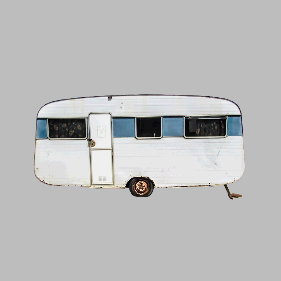

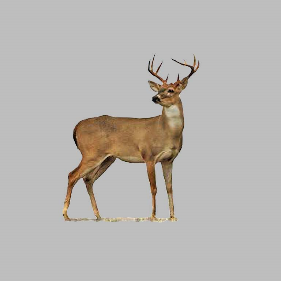

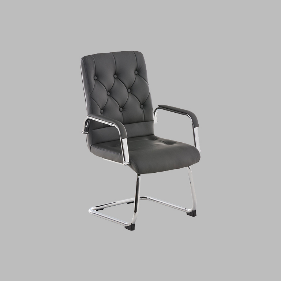

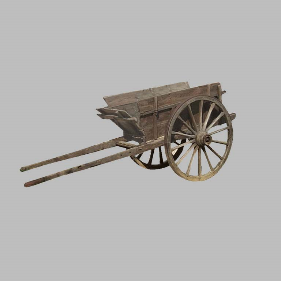


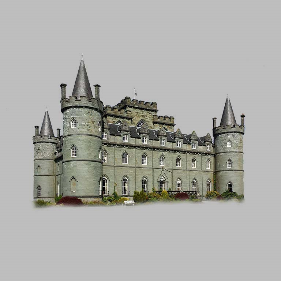

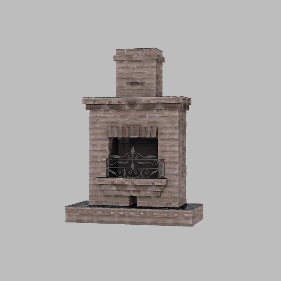

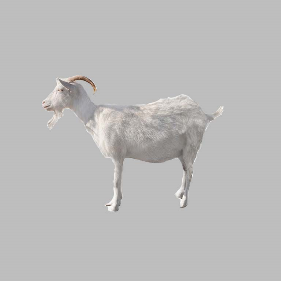

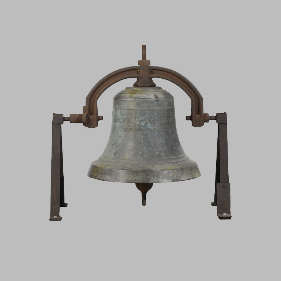


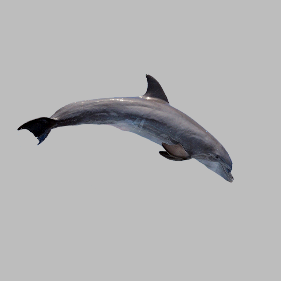

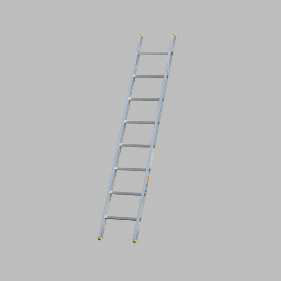

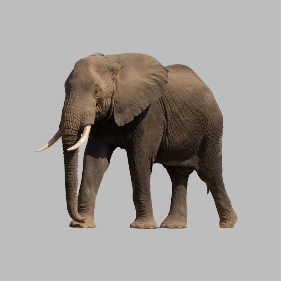

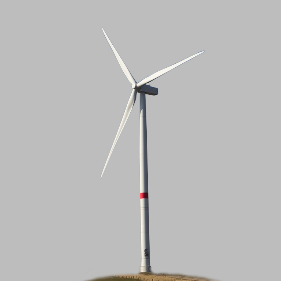


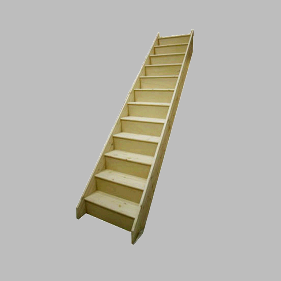

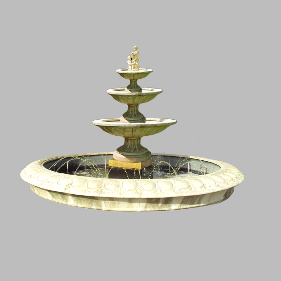

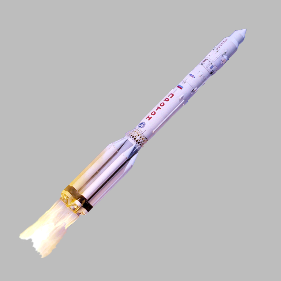

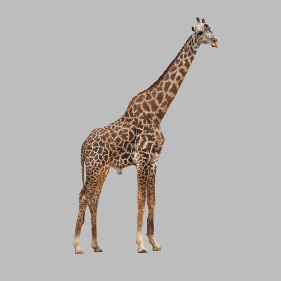


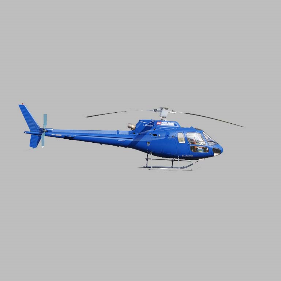

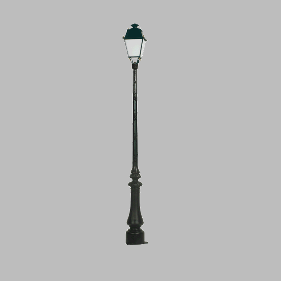

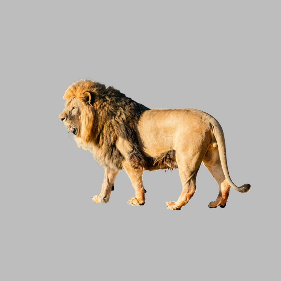

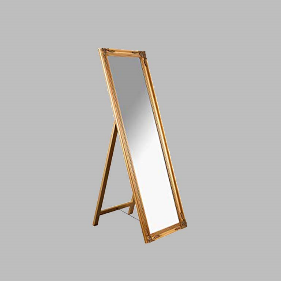

Supplement: S1 Appendix — (DOCX) [file pone.0248239.s001.docx]
