# Supplementary material for: Short-term upper limb immobilization and the embodied view of memory: A pilot study
Source: PLoS One. 2021 Mar 11;16(3):e0248239. doi: 10.1371/journal.pone.0248239 (PMC7951805; doi:10.1371/journal.pone.0248239)
Supplement: S2 Appendix — (DOCX) [file pone.0248239.s002.docx]

S2 Appendix

Items’ names and characteristics

**Graspable objects**

| **Name (French)** | **Name (English)** | **Lexical frequency** | **Number of phonemes** | **Object type** | **List** |
| --- | --- | --- | --- | --- | --- |
| allumette | match | 15,65 | 6 | graspable | Graspable 1 |
| balai | broom | 10,91 | 4 | graspable | Graspable 1 |
| biberon | baby bottle | 6,65 | 6 | graspable | Graspable 1 |
| bouée | buoy | 2,04 | 3 | graspable | Graspable 1 |
| briquet | lighter | 10,64 | 5 | graspable | Graspable 1 |
| cafetière | coffee pot | 2,07 | 8 | graspable | Graspable 1 |
| canne | stick | 10,91 | 3 | graspable | Graspable 1 |
| carotte | carrot | 7,12 | 5 | graspable | Graspable 1 |
| casserole | pot | 4,95 | 7 | graspable | Graspable 1 |
| chargeur | battery charger | 4,63 | 6 | graspable | Graspable 1 |
| ciseaux | scissors | 8,72 | 4 | graspable | Graspable 1 |
| clé | key | 118,13 | 3 | graspable | Graspable 1 |
| compas | compas | 1,5 | 4 | graspable | Graspable 1 |
| couteau | knife | 58,15 | 4 | graspable | Graspable 1 |
| éponge | sponge | 7,11 | 4 | graspable | Graspable 1 |
| fleur | flower | 99,75 | 4 | graspable | Graspable 1 |
| fourchette | fork | 5,85 | 6 | graspable | Graspable 1 |
| marteau | hammer | 12,63 | 5 | graspable | Graspable 1 |
| oignon | onion | 19,98 | 3 | graspable | Graspable 1 |
| orange | orange | 16,29 | 4 | graspable | Graspable 1 |
| panier | basket | 15,72 | 5 | graspable | Graspable 1 |
| parapluie | umbrella | 7,38 | 8 | graspable | Graspable 1 |
| peigne | comb | 6,81 | 3 | graspable | Graspable 1 |
| pelle | shovel | 10,1 | 3 | graspable | Graspable 1 |
| perceuse | drill | 1,22 | 6 | graspable | Graspable 1 |
| pile | battery | 21,3 | 3 | graspable | Graspable 1 |
| pinceau | paintbrush | 4,59 | 4 | graspable | Graspable 1 |
| pistolet | handgun | 34,92 | 7 | graspable | Graspable 1 |
| scie | saw | 5,36 | 2 | graspable | Graspable 1 |
| seau | bucket | 9,01 | 2 | graspable | Graspable 1 |
| tasse | mug | 21,89 | 3 | graspable | Graspable 1 |
| téléphone | phone | 160,8 | 7 | graspable | Graspable 1 |
| agrafeuse | stapler | 0,79 | 7 | graspable | Graspable 2 |
| ampoule | bulb | 7,66 | 4 | graspable | Graspable 2 |
| banane | banana | 11,14 | 5 | graspable | Graspable 2 |
| boîte | box | 88,81 | 4 | graspable | Graspable 2 |
| bouteille | bottle | 57,24 | 5 | graspable | Graspable 2 |
| brosse | hairbrush | 8,43 | 4 | graspable | Graspable 2 |
| cadenas | lock | 2,1 | 6 | graspable | Graspable 2 |
| carnet | notebook | 13,23 | 5 | graspable | Graspable 2 |
| citron | lemon | 10,92 | 5 | graspable | Graspable 2 |
| cuillère | spoon | 7,3 | 6 | graspable | Graspable 2 |
| cutter | cutter | 2,41 | 5 | graspable | Graspable 2 |
| épée | sword | 32,81 | 3 | graspable | Graspable 2 |
| flûte | flute | 10,13 | 4 | graspable | Graspable 2 |
| fraise | strawberry | 12 | 4 | graspable | Graspable 2 |
| gomme | eraser | 3,81 | 3 | graspable | Graspable 2 |
| hache | axe | 10,23 | 2 | graspable | Graspable 2 |
| menotte | handcuffs | 11,65 | 5 | graspable | Graspable 2 |
| montre | watch | 48,4 | 4 | graspable | Graspable 2 |
| noix | walnut | 12,83 | 3 | graspable | Graspable 2 |
| passoire | sieve | 1,44 | 6 | graspable | Graspable 2 |
| pince | clamp | 9 | 3 | graspable | Graspable 2 |
| plume | feather | 15,29 | 4 | graspable | Graspable 2 |
| poêle | frying pan | 5,12 | 4 | graspable | Graspable 2 |
| poignée | door handle | 13,61 | 5 | graspable | Graspable 2 |
| pomme | apple | 42,35 | 3 | graspable | Graspable 2 |
| raquette | racket | 2,05 | 5 | graspable | Graspable 2 |
| rasoir | razor | 8,92 | 6 | graspable | Graspable 2 |
| savon | soap | 16,68 | 4 | graspable | Graspable 2 |
| sifflet | whistle | 4,61 | 5 | graspable | Graspable 2 |
| stylo | pencil | 17,73 | 5 | graspable | Graspable 2 |
| thermomètre | thermometer | 1,43 | 9 | graspable | Graspable 2 |
| tournevis | screwdriver | 3,46 | 8 | graspable | Graspable 2 |

**Non-graspable objects**

| **Name (French)** | **Name (english)** | **Lexical frequency** | **Number of phonemes** | **Object type** | **list** |
| --- | --- | --- | --- | --- | --- |
| arbre | tree | 81,69 | 4 | Non-graspable | Non-graspable 1 |
| armoire | closet | 9,79 | 6 | Non-graspable | Non-graspable 1 |
| avion | plane | 128,35 | 4 | Non-graspable | Non-graspable 1 |
| banc | bench | 10,76 | 2 | Non-graspable | Non-graspable 1 |
| billard | pool table | 7,84 | 5 | Non-graspable | Non-graspable 1 |
| cactus | cactus | 2,86 | 6 | Non-graspable | Non-graspable 1 |
| canapé | sofa | 18,58 | 6 | Non-graspable | Non-graspable 1 |
| canon | cannon | 22,22 | 4 | Non-graspable | Non-graspable 1 |
| caravane | caravan | 11,64 | 7 | Non-graspable | Non-graspable 1 |
| cerf | deer | 7,56 | 3 | Non-graspable | Non-graspable 1 |
| chaise | chair | 40,02 | 3 | Non-graspable | Non-graspable 1 |
| charrette | cart | 6,98 | 5 | Non-graspable | Non-graspable 1 |
| cheminée | chimney | 11,39 | 6 | Non-graspable | Non-graspable 1 |
| dauphin | dolphin | 4,71 | 4 | Non-graspable | Non-graspable 1 |
| échelle | scale | 14,09 | 4 | Non-graspable | Non-graspable 1 |
| éolienne | wind turbine | 0,14 | 6 | Non-graspable | Non-graspable 1 |
| escalier | stair | 32,29 | 7 | Non-graspable | Non-graspable 1 |
| girafe | giraffe | 3,5 | 5 | Non-graspable | Non-graspable 1 |
| hélicoptère | helicopter | 13,96 | 9 | Non-graspable | Non-graspable 1 |
| lion | lion | 20,86 | 3 | Non-graspable | Non-graspable 1 |
| miroir | mirror | 28,35 | 6 | Non-graspable | Non-graspable 1 |
| moto | motorcycle | 25,23 | 4 | Non-graspable | Non-graspable 1 |
| pieuvre | octopus | 1,73 | 5 | Non-graspable | Non-graspable 1 |
| pneu | tyre | 13,44 | 3 | Non-graspable | Non-graspable 1 |
| renard | fox | 6,66 | 5 | Non-graspable | Non-graspable 1 |
| singe | monkey | 35,48 | 3 | Non-graspable | Non-graspable 1 |
| squelette | skeleton | 6,75 | 6 | Non-graspable | Non-graspable 1 |
| tableau | blackboard | 50,11 | 5 | Non-graspable | Non-graspable 1 |
| toboggan | toboggan | 0,86 | 6 | Non-graspable | Non-graspable 1 |
| tonneau | barrel | 4,37 | 4 | Non-graspable | Non-graspable 1 |
| trampoline | trampoline | 1,17 | 8 | Non-graspable | Non-graspable 1 |
| ventilateur | fan | 3,02 | 9 | Non-graspable | Non-graspable 1 |
| armure | armour | 5,46 | 5 | Non-graspable | Non-graspable 2 |
| autruche | ostrich | 3,53 | 5 | Non-graspable | Non-graspable 2 |
| baignoire | bathtumb | 12,39 | 6 | Non-graspable | Non-graspable 2 |
| barbecue | barbecue | 5,87 | 8 | Non-graspable | Non-graspable 2 |
| barrière | barrier | 9,13 | 6 | Non-graspable | Non-graspable 2 |
| bateau | boat | 124,82 | 4 | Non-graspable | Non-graspable 2 |
| batterie | drums | 14,24 | 6 | Non-graspable | Non-graspable 2 |
| camion | truck | 59,46 | 5 | Non-graspable | Non-graspable 2 |
| château | castle | 43,68 | 4 | Non-graspable | Non-graspable 2 |
| chèvre | goat | 14,08 | 4 | Non-graspable | Non-graspable 2 |
| cloche | bell | 19,72 | 4 | Non-graspable | Non-graspable 2 |
| éléphant | elephant | 15,36 | 5 | Non-graspable | Non-graspable 2 |
| fontaine | fountain | 7,72 | 5 | Non-graspable | Non-graspable 2 |
| fusée | rocket | 10,09 | 4 | Non-graspable | Non-graspable 2 |
| lampadaire | lamp post | 1,89 | 7 | Non-graspable | Non-graspable 2 |
| montgolfière | balloon | 0,81 | 9 | Non-graspable | Non-graspable 2 |
| niche | kennel | 2,27 | 3 | Non-graspable | Non-graspable 2 |
| ours | bear | 24,57 | 3 | Non-graspable | Non-graspable 2 |
| palette | pallet | 0,87 | 5 | Non-graspable | Non-graspable 2 |
| piano | piano | 22,22 | 5 | Non-graspable | Non-graspable 2 |
| piscine | swimming pool | 23,62 | 5 | Non-graspable | Non-graspable 2 |
| poubelle | bin | 21,34 | 5 | Non-graspable | Non-graspable 2 |
| puits | wells | 19,52 | 3 | Non-graspable | Non-graspable 2 |
| radiateur | radiator | 3,65 | 8 | Non-graspable | Non-graspable 2 |
| statue | statue | 19,5 | 5 | Non-graspable | Non-graspable 2 |
| télévision | television | 26,38 | 9 | Non-graspable | Non-graspable 2 |
| tente | tent | 16,65 | 3 | Non-graspable | Non-graspable 2 |
| toilette | toilet | 62,84 | 6 | Non-graspable | Non-graspable 2 |
| tondeuse | mower | 2,04 | 5 | Non-graspable | Non-graspable 2 |
| tracteur | tractor | 3,86 | 7 | Non-graspable | Non-graspable 2 |
| vache | cow | 47,71 | 3 | Non-graspable | Non-graspable 2 |
| zèbre | zebra | 3,8 | 4 | Non-graspable | Non-graspable 2 |
